# A TatABC-Type Tat Translocase Is Required for Unimpaired Aerobic Growth of *Corynebacterium glutamicum* ATCC13032

**DOI:** 10.1371/journal.pone.0123413

**Published:** 2015-04-02

**Authors:** Dan Oertel, Sabrina Schmitz, Roland Freudl

**Affiliations:** Institut für Bio- und Geowissenschaften 1, IBG1: Biotechnologie, Forschungszentrum Jülich GmbH, Jülich, Germany; INRA Clermont-Ferrand Research Center, FRANCE

## Abstract

The twin-arginine translocation (Tat) system transports folded proteins across the cytoplasmic membrane of bacteria and the thylakoid membrane of plant chloroplasts. *Escherichia coli* and other Gram-negative bacteria possess a TatABC-type Tat translocase in which each of the three inner membrane proteins TatA, TatB, and TatC performs a mechanistically distinct function. In contrast, low-GC Gram-positive bacteria, such as *Bacillus subtilis*, use a TatAC-type minimal Tat translocase in which the TatB function is carried out by a bifunctional TatA. In high-GC Gram-positive Actinobacteria, such as *Mycobacterium tuberculosis* and *Corynebacterium glutamicum*, *tatA*, *tatB*, and *tatC* genes can be identified, suggesting that these organisms, just like *E*. *coli*, might use TatABC-type Tat translocases as well. However, since contrary to this view a previous study has suggested that *C*. *glutamicum* might in fact use a TatAC translocase with TatB only playing a minor role, we reexamined the requirement of TatB for Tat-dependent protein translocation in this microorganism. Under aerobic conditions, the misassembly of the Rieske iron-sulfur protein QcrA was identified as a major reason for the severe growth defect of Tat-defective *C*. *glutamicum* mutant strains. Furthermore, our results clearly show that TatB, besides TatA and TatC, is strictly required for unimpaired aerobic growth. In addition, TatB was also found to be essential for the secretion of a heterologous Tat-dependent model protein into the *C*. *glutamicum* culture supernatant. Together with our finding that expression of the *C*. *glutamicum* TatB in an *E*. *coli* Δ*tatB* mutant strain resulted in the formation of an active Tat translocase, our results clearly indicate that a TatABC translocase is used as the physiologically relevant functional unit for Tat-dependent protein translocation in *C*. *glutamicum* and, most likely, also in other TatB-containing Actinobacteria.

## Introduction

The transport of proteins into or across biological membranes is catalyzed by membrane-bound, multi-component protein translocases. In most bacteria, the major route of protein export is the general secretion (Sec) pathway [1] that translocates its substrates as unfolded polypeptide chains [2]. In contrast, the alternative twin-arginine translocation (Tat) pathway transports fully folded proteins across the membrane [3]. Tat substrates are often proteins that have to recruit a cofactor in the cytosol and, therefore, must acquire their folded status prior to their export [4]. The signal peptides of Tat substrates contain a highly conserved twin-arginine motif (S/T-**R-R**-X-F-L-K) that has been shown to be important for the productive recognition and binding of the Tat substrates by the Tat translocase [5–7].

The Tat pathway has been most extensively studied in *Escherichia coli* and, in this organism (and also most other diderm-lipopolysaccharide (LPS) [8–10] Gram-negative bacteria), the respective Tat translocase is a TatABC-type translocase consisting of three inner membrane proteins, TatA, TatB, and TatC, that jointly are expressed from an operon [11]. Another *tat* gene, *tatE*, is located elsewhere in the genome of *E*. *coli* and encodes a paralogue of TatA. Due to the fact that *tatE* is expressed at a level 50–200 times lower than *tatA* [12], *tatE* is commonly regarded as a cryptic gene duplication of *tatA*. After folding and, if required, cofactor insertion, proteins containing a Tat signal peptide are recognized by a substrate receptor complex consisting of TatB and TatC [7, 13]. Subsequently, homo-oligomers of TatA are recruited to the substrate-loaded receptor complex in a proton motive force (pmf)-dependent manner [14], followed by substrate translocation across the inner membrane in a step that is still poorly understood. Whereas some models propose that TatA multimers form a substrate-fitted protein-conducting channel (e.g. [15]), an alternative model suggests that the TatA multimers might lead to a punctual weakening of the membrane in the vicinity of the TatBC-substrate complex and thereby allows a TatC-driven translocation of the substrate without the need of an actual translocation channel [16]. In TatABC-type Tat translocases, TatA and TatB carry out distinct functions and cannot substitute for each other [17].

A different scenario is found in monoderm [8–10] Gram-positive bacteria with low-GC-content genomes, such as the well-studied model organism *Bacillus subtilis*. In these bacteria, TatB is lacking and Tat-dependent protein translocation is performed by TatAC-type minimal Tat translocases in which the TatB function is carried out by a bifunctional TatA subunit [18–22]. However, in Gram-positive bacteria with high-GC-content genomes such as members of the phylum Actinobacteria (as exemplified by the monoderm [8–10, 23] bacteria *Streptomyces coelicolor* and *Streptomyces lividans*, and the diderm-mycolate [8–10, 23] bacteria *Mycobacterium tuberculosis* and *Corynebacterium glutamicum*), genes encoding homologues of TatA, TatB, TatC, (and also sometimes TatE) can be found. A notable difference to the situation in *E*. *coli* and other Gram-negative bacteria with TatABC-type Tat translocases, where the three Tat components are expressed from a *tatABC* operon, is the finding that the *tat* genes in the Gram-positive bacteria with high-GC-content genomes are differently organized. Here, only *tatA* and *tatC* are present in an operon and, in contrast to the situation mentioned above, the *tatB* gene is located elsewhere on the chromosome [24]. Nevertheless, the presence of all three *tat* genes in the respective genomes suggests that this class of bacteria, like *E*. *coli*, might use TatABC-type translocases as functional units of Tat-dependent protein translocation. A strong support for this view comes from investigations of the Tat system of *M*. *tuberculosis* which, in contrast to the Tat system of most other organisms tested so far, is essential for viability. Disruption of *tatA*, *tatC*, and importantly also of *tatB* on the chromosome of *M*. *tuberculosis* was not possible, strongly indicating that each Tat component is of equal importance (as typically is the case for TatABC-type Tat translocases) and arguing against the possibility of a TatAC translocase being the functional unit of the Tat system in this microorganism [25]. In line with these findings, results from the Actinobacteria *S*. *lividans* [26–27] and *S*. *coelicolor* [28–29] also showed that *tatB* mutants of these microorganisms are similarly affected in Tat-dependent protein translocation as their respective *tatA* or *tatC* mutant strains. A somewhat different situation was described by Kikuchi et al. [30] for the Tat system of *C*. *glutamicum*. Also in *C*. *glutamicum*, a *tatAC* operon and a *tatB* gene located at different positions on the chromosome can be identified. Additionally, *C*. *glutamicum* also possesses a *tatE* gene located at another position in the genome that, like the situation in *E*. *coli*, presumably is a cryptic gene duplication of *tatA* [30]. The Tat system is not absolutely essential for the viability of *C*. *glutamicum* since all *tat* genes can be deleted. However, Δ*tatA* and Δ*tatC* mutant strains show drastic growth defects compared to the wild-type strain, indicating that the mislocalization of a physiologically important Tat substrate is responsible for this phenotype. Deletion of *tatE* had no noticeable effect on the growth of *C*. *glutamicum*, supporting the view that TatE is functionally redundant to TatA and does not play a significant role also in this microorganism. Strikingly however and in stark contrast to the Δ*tatA* and Δ*tatC* mutant strains, growth of a Δ*tatB* deletion mutant was described as being almost identical to that of the *tat* wild-type strain. Based on these findings it was concluded that TatB is dispensable and that *C*. *glutamicum* uses a TatAC-type minimal translocase as the basic functional unit for Tat-dependent protein translocation [30].

The major branch of the aerobic respiratory chain of *C*. *glutamicum* involves the cytochrome *bc*_*1*_ *-aa*_*3*_ super-complex that includes the Rieske iron-sulfur component QcrA [31]. In Actinobacteria, the Rieske proteins are polytopic integral membrane proteins with three transmembrane segments. For the Rieske protein of *S*. *coelicolor*, it has been shown that the Sec and the Tat translocases cooperate in the assembly process [32]. The amino-terminal domain encompassing the first two transmembrane segments is integrated into the cytoplasmic membrane via the Sec system. Subsequently, the third transmembrane segment which corresponds to a non-cleavable internal Tat signal peptide mediates the Tat-dependent translocation of the fully folded carboxyl-terminal iron-sulfur cofactor-containing domain to the *trans*-side of the cytoplasmic membrane [32–33]. Deletion of the genes encoding the cytochrome *bc*_*1*_ complex (i.e. *qcrABC*) causes a severe growth defect of *C*. *glutamicum* under aerobic conditions [31, 34]. It is therefore a likely possibility that the similarly severe growth defect caused by the absence of a functional Tat system might in fact be due to the mislocalization of the iron-sulfur domain of the Rieske protein QcrA.

Since the results described for the Actinobacteria *M*. *tuberculosis*, *S*. *lividans* and *S*. *coelicolor* are contradictory to the results described for *C*. *glutamicum* with respect to the suggested types of Tat translocases that operate in the respective organisms, we reexamined the role of TatB in the Tat-dependent protein translocation in *C*. *glutamicum* with a focus on the proper localization of the Rieske iron-sulfur protein QcrA. Evidence is presented that the mislocalization of QcrA is in fact a major reason for the growth defects of *C*. *glutamicum* strains defective in Tat-dependent protein translocation. Furthermore, our results make it very likely that, besides TatA and TatC, also TatB is required for efficient QcrA assembly and, as a consequence, for normal growth. In addition, the presence of TatB was found to be essential for the secretion of a heterologous Tat-dependent model protein. Taken together, our combined results clearly indicate that a TatABC-type translocase is the functional unit for Tat-dependent protein translocation in *C*. *glutamicum* and, most likely, also in other TatB-containing Actinobacteria.

## Results

### Deletion of *tatAC*, *tatA/E*, or *tatB* abolishes Tat-dependent protein translocation and causes a severe growth defect in *C*. *glutamicum*

In TatA(E)BC-type translocases, as exemplified by the *E*. *coli* Tat translocase, TatA(E), TatB, and TatC perform distinct mechanistically relevant functions and cannot substitute for each other. Mutant strains possessing deletions in either of the corresponding *tat* genes are therefore expected to possess similar phenotypes that ultimately are caused by a non-functional Tat export machinery. To gain more insight in the type of Tat translocase that is used in the Actinobacterium *C*. *glutamicum*, we compared different *tat* deletion mutants with respect to their growth phenotypes and their ability to translocate a Tat-dependent model protein across the cytoplasmic membrane. Besides the previously described Δ*tatAC* mutant strain [35], a Δ*tatB* and a Δ*tatA/E* mutant were additionally constructed as described in the Materials and Methods section.

**Fig 1:**
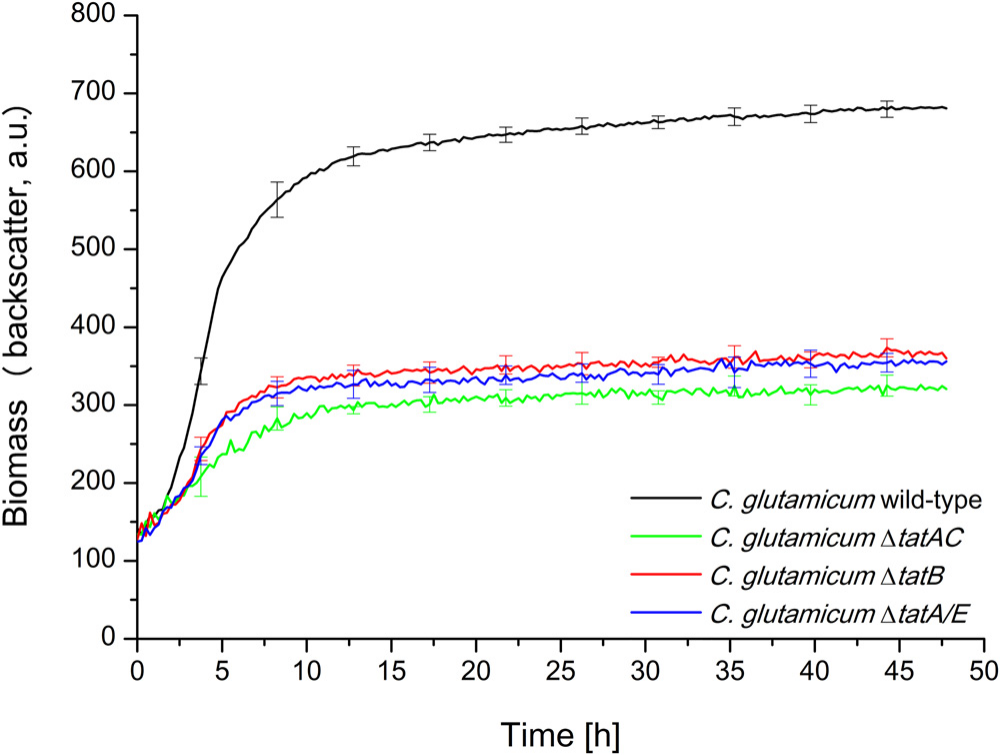
Growth phenotype of *C*. *glutamicum* ATCC13032 wild-type, Δ*tatAC*, Δ*tatA/E*, and Δ*tatB* mutants. Cells were inoculated to an OD_600_ of 0.5 in 750 μl BHIS medium and cultivated in 48-well FlowerPlates in a BioLector system for 48 h at 30°C, 1100 rpm under constant 85% relative humidity. Growth of the respective *C*. *glutamicum* strains, as indicated in the figure, was monitored as backscattered light (620 nm; signal gain factor 20) in 15 min intervals. The growth curves show one representative experiment of three independent biological replicates. Standard deviations are given for 10 selected time points. a.u.: arbitrary units.

**Fig 2:**
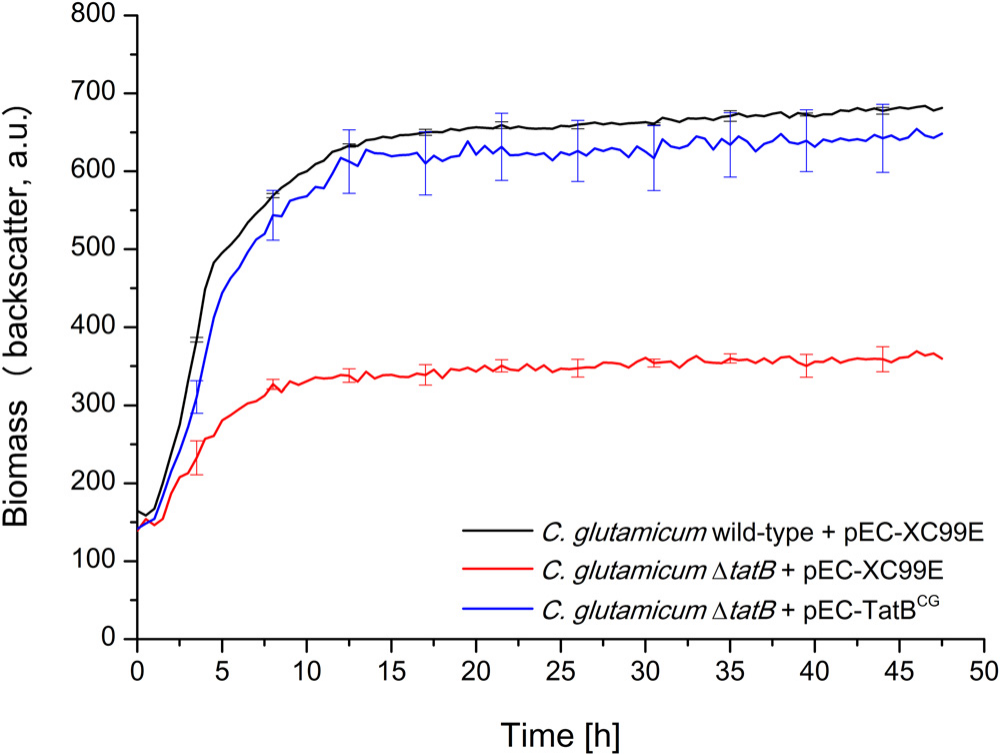
The growth defect of the *C*. *glutamicum* Δ*tatB* mutant is reversed by expressing the *C*. *glutamicum* TatB *in trans* from plasmid pEC-TatB^CG^. Cells were inoculated to an OD_600_ of 0.5 in 750 μl BHIS medium containing 10 μM IPTG and cultivated in 48-well FlowerPlates in a BioLector system for 48 h at 30°C, 1100 rpm under constant 85% relative humidity. Growth of the respective *C*. *glutamicum* strains, as indicated in the figure, was monitored as backscattered light (620 nm; signal gain factor 20) in 15 min intervals. The growth curves show one representative experiment of three independent biological replicates. Standard deviations are given for 10 selected time points. a.u.: arbitrary units.

First, growth of *C*. *glutamicum* wild-type under aerobic conditions in BHIS medium was compared to the growth of Δ*tatAC*, Δ*tatA/E*, and Δ*tatB* mutant strains in 48-well FlowerPlates using a BioLector micro reactor cultivation device. In the BioLector, a high oxygen transfer rate and therefore growth under fully aerobic conditions can be achieved. Furthermore, in this device, the growth of bacterial strains can be determined online via light scatter measurements, thereby allowing a real time monitoring of biomass formation in each of the 48 growth chambers of the FlowerPlate [36]. As shown in Fig 1, the Δ*tatAC* and Δ*tatA/E* mutant strains showed severe growth defects when compared to the unaltered wild-type strain, being fully in line with the results published by Kikuchi et al. [30] for Δ*tatA* and Δ*tatC* mutant strains. Strikingly however and in stark contrast to the previously published findings [30], we observed an identically severe growth defect also for the Δ*tatB* mutant strain. The growth defect of the Δ*tatB* mutant could almost completely be reverted by expressing TatB *in trans* from plasmid pEC-TatB^CG^, but not by introducing the pEC-X99E empty vector as control (Fig 2), confirming that the growth defect of the Δ*tatB* strain is indeed caused by the lack of TatB and not due to effects caused by the *tatB* deletion on the neighboring genes on the chromosome.

**Fig 3:**
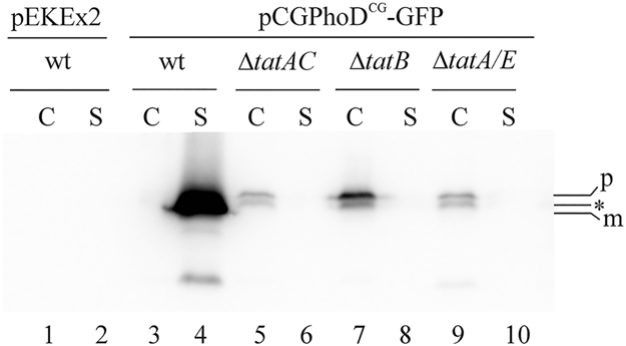
Analysis of PhoD^CG^-GFP secretion in *C*. *glutamicum* wild-type and *tat* mutant strains. Cultures of *C*. *glutamicum* strains expressing the Tat-dependent PhoD^CG^-GFP model protein [35] were fractionated into cells (C) and supernatant (S). Samples of the fractions corresponding to an equal number of cells (i.e. an OD_600_ of 1.0) were subjected to SDS-PAGE and immunoblotting using GFP-specific antibodies. The following strains were analyzed: *C*. *glutamicum* wild-type (wt) containing the empty vector pEKEx2 as negative control (lanes 1 and 2), *C*. *glutamicum* wild-type (wt) containing plasmid pCGPhoD^CG^-GFP (lanes 3 and 4), and the pCGPhoD^CG^-GFP-containing *C*. *glutamicum* mutant strains Δ*tatAC* (lanes 5 and 6), Δ*tatB* (lanes 7 and 8), and Δ*tatA/E* (lanes 9 and 10). p: PhoD^CG^-GFP precursor; asterisk: cytosolic degradation product; m: mature-sized GFP protein.

Next, Tat-dependent protein translocation was analyzed using a previously described model protein (PhoD^CG^-GFP [35]) consisting of the signal peptide of the *C*. *glutamicum* Tat substrate PhoD (an alkaline phosphatase) fused to green fluorescent protein (GFP). As shown in Fig 3, large amounts of mature GFP are present in the supernatant of *C*. *glutamicum* wild-type containing plasmid pCGPhoD^CG^-GFP (lane 4). Furthermore, hardly any detectable GFP-derived polypeptides were present in the cellular fraction of the same strain (lane 3). In contrast, no secreted GFP protein is found in the supernatant of the Δ*tatAC*, Δ*tatA/E*, or Δ*tatB* mutant strains (lanes 6, 8, 10) and, in the cell fractions of the corresponding strains (lanes 5, 7, 9), an accumulation of PhoD^CG^-GFP precursor and degradation products of it can be observed. These results clearly indicate that TatA, TatB, and TatC are equally important for the translocation of PhoD^CG^-GFP across the cytoplasmic membrane of *C*. *glutamicum*.

### The Rieske iron-sulfur protein QcrA is an important Tat substrate in *C*. *glutamicum*

The Rieske iron-sulfur protein QcrA is a component of the cytochrome *bc*_*1*_ *-aa*_*3*_ super-complex which constitutes the major branch of the aerobic respiratory chain of *C*. *glutamicum* [31]. Like other actinobacterial Rieske proteins, the *C*. *glutamicum* QcrA protein possesses three transmembrane segments. Preceding the third transmembrane segment, an internal Tat consensus motif (G-**RR**-K-L-I) can be identified suggesting that, like the situation in *S*. *coelicolor* [32], the C-terminal iron-sulfur cluster-containing domain might be translocated to the *trans*-side of the cytoplasmic membrane in a Tat-dependent manner. Furthermore, since deletion of the genes encoding the cytochrome *bc*_*1*_ complex (including *qcrA*) causes a severe growth defect to *C*. *glutamicum* [37], we thought that it is a likely possibility that the severe growth defect of the *C*. *glutamicum tat* mutants is in fact caused by the mislocalization of QcrA.

**Fig 4:**
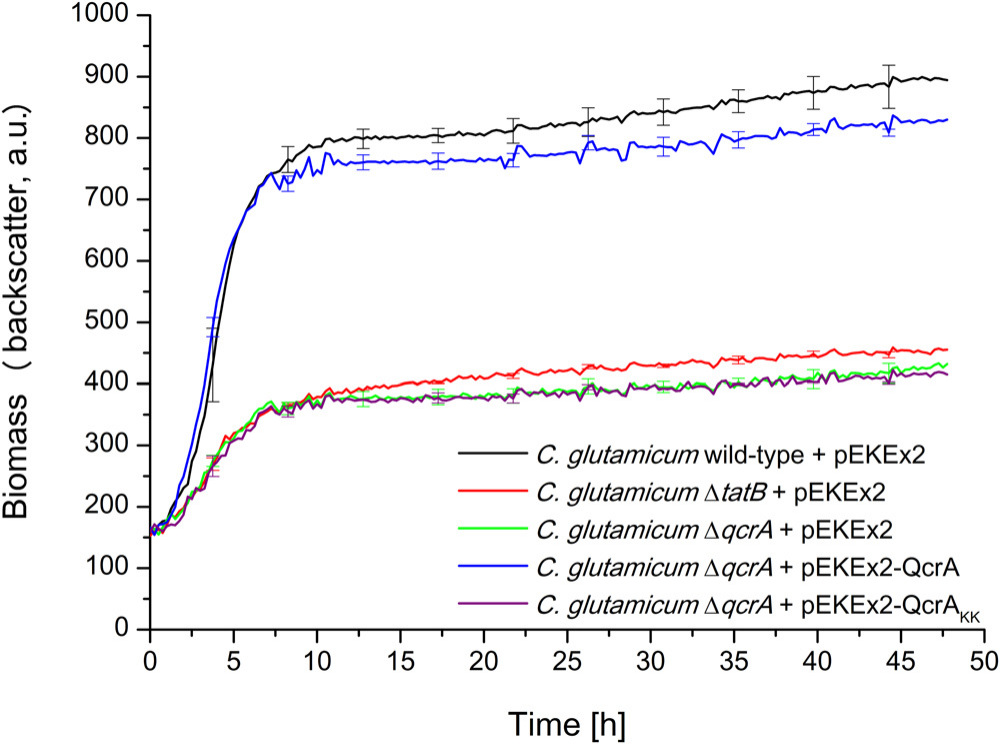
The correct membrane assembly of the physiologically important Tat substrate QcrA is required for unimpaired growth of *C*. *glutamicum*. Cells were inoculated to an OD_600_ of 0.5 in 750 μl BHIS medium containing 100 μM IPTG and cultivated in 48-well FlowerPlates in a BioLector system for 48 h at 30°C, 1100 rpm under constant 85% relative humidity. Growth of the respective *C*. *glutamicum* strains, as indicated in the figure, was monitored as backscattered light (620 nm; signal gain factor 20) in 15 min intervals. The growth curves show one representative experiment of three independent biological replicates. Standard deviations are given for 10 selected time points. a.u.: arbitrary units.

To experimentally address these assumptions, we first compared the growth of a *C*. *glutamicum* wild-type strain possessing the pEKEx2 empty vector with the growth of pEKEx2-containing mutant strains *C*. *glutamicum* Δ*qcrA* and *C*. *glutamicum* Δ*tatB* using the BioLector micro-reactor cultivation device. As shown in Fig 4, compared to *C*. *glutamicum* wild-type, both mutant strains show a severely impaired growth under aerobic conditions. Notably, the growth behavior of both mutant strains is almost identical, a finding that would be consistent with mislocalization of QcrA being the actual cause for the growth defect associated with *C*. *glutamicum tat* mutant strains.

To directly show that the correct localization of the QcrA iron-sulfur domain requires the Tat translocase, *C*. *glutamicum* Δ*qcrA* was transformed with plasmids pEKEx2-QcrA (encoding unaltered QcrA), or pEKEx2-QcrA_KK_ (encoding a mutated QcrA protein in which the two conserved twin arginine residues of the Tat-motif in front of the third transmembrane segment are replaced by a twin lysine pair). Replacement of the twin-arginines by a pair of lysines has been shown previously to significantly reduce or even completely abolish Tat-dependent translocation for a variety of different Tat substrates [38–39]. As shown in Fig 4, the growth defect of the Δ*qcrA* mutant could be almost completely reverted by expressing unaltered QcrA *in trans* from plasmid pEKEx2-QcrA, but not by the introduction of the pEKEx2 empty vector as the negative control. Importantly, no reversion of the growth defect of *C*. *glutamicum* Δ*qcrA* was observed when the QcrA_KK_ variant was expressed from plasmid pEKEx2-QcrA_KK_, indicating that the Tat system is involved in the translocation of the iron-sulfur domain of the Rieske iron sulfur protein QcrA also in *C*. *glutamicum*.

**Fig 5:**
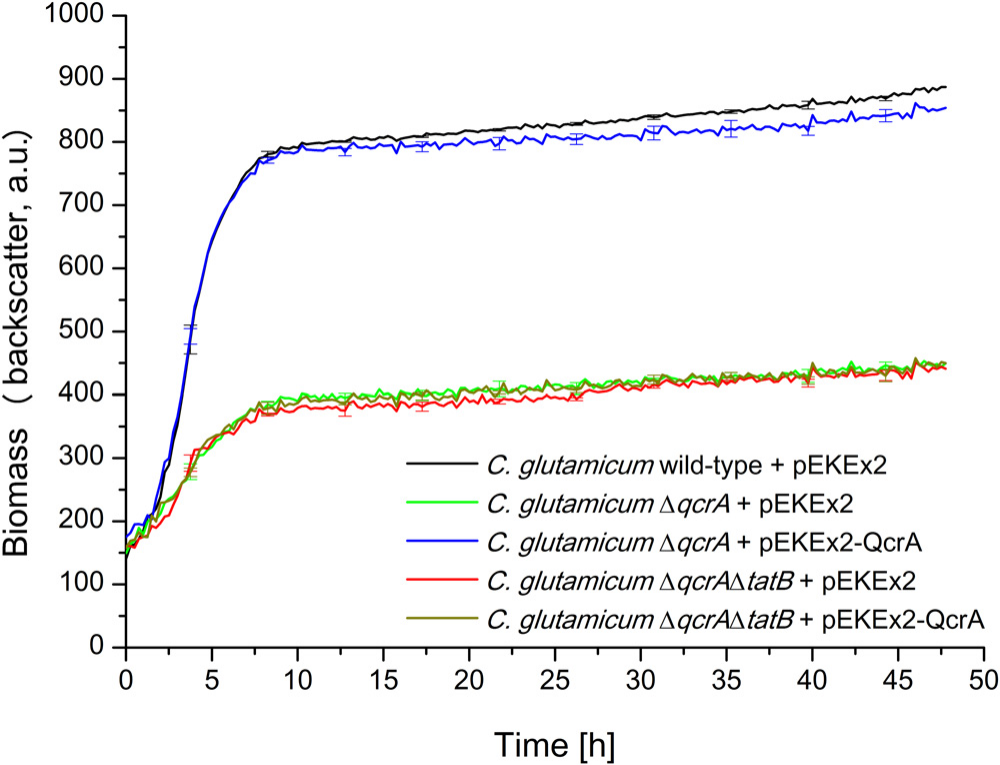
The growth defect of a *C*. *glutamicum* Δ*qcrA* Δ*tatB* double mutant cannot be complemented *in trans* by plasmid-encoded QcrA. Cells were inoculated to an OD_600_ of 0.5 in 750 μl BHIS medium containing 100 μM IPTG and cultivated in 48-well FlowerPlates in a BioLector system for 48 h at 30°C, 1100 rpm under constant 85% relative humidity. Growth of the respective *C*. *glutamicum* strains, as indicated in the figure, was monitored as backscattered light (620 nm; signal gain factor 20) in 15 min intervals. The growth curves show one representative experiment of three independent biological replicates. Standard deviations are given for 10 selected time points. a.u.: arbitrary units.

As described in the previous paragraph, the growth defect of a *C*. *glutamicum* Δ*qcrA* mutant could almost completely be reverted by *in trans* expression of plasmid-encoded QcrA. Next, we asked whether or not such an *in trans* complementation of the *qcrA* deletion in the chromosome can still be observed when the TatB component is missing. For this, a Δ*qcrA* Δ*tatB* double mutant was constructed and transformed with plasmid pEKEx2-QcrA or the pEKEx2 empty vector, respectively. In contrast to the situation found for the Δ*qcrA* single mutant strain, no reversion of the growth defect was observed in the Δ*qcrA* Δ*tatB* double mutant when QcrA was expressed from pEKEx2-QcrA *in trans* (Fig 5). One feasible, and in our opinion the most likely explanation for the observed results is that the presence of TatB is required for the correct membrane assembly of QcrA in *C*. *glutamicum*. However, at present we cannot completely rule out the possibility that, besides QcrA, one or more additional Tat substrate(s) exist whose mislocalization together has an identically severe negative impact on the growth of *C*. *glutamicum* as the mislocalization of QcrA alone and, if so, that TatB could specifically function in the correct localization of these additional proteins. In any case, our results nevertheless clearly demonstrate that the presence of TatB is of crucial importance for the correct localization of at least one physiologically important Tat substrate and, as a consequence, for unimpaired growth of *C*. *glutamicum* under aerobic conditions.

### Heterologous expression of the *C*. *glutamicum* TatB in *E*. *coli tat* mutant strains

**Fig 6:**
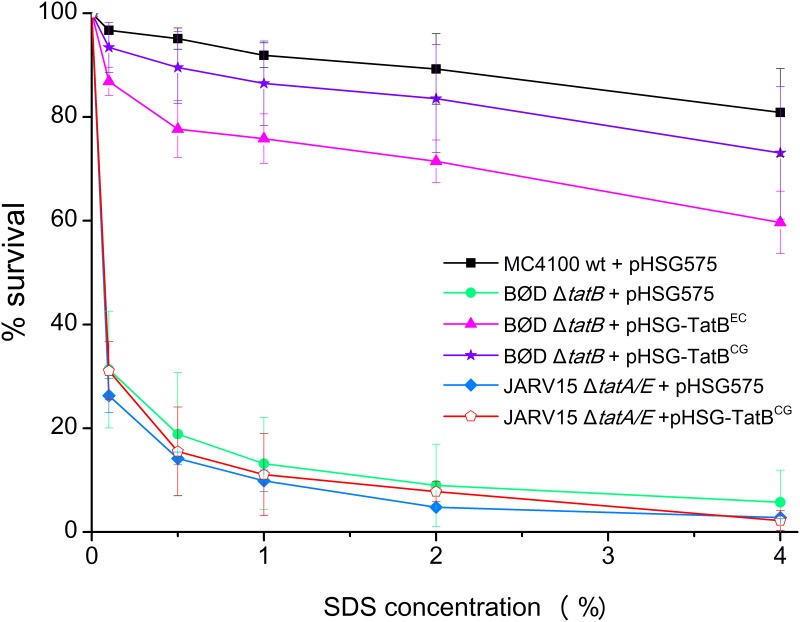
SDS sensitivity of *E*. *coli tat* mutant strains expressing the *C*. *glutamicum* TatB protein from plasmid pHSG-TatB^CG^. MC4100 (wild-type) containing the empty vector pHSG575 (black squares), BØD (Δ*tatB*) containing pHSG575 (green circles), BØD containing pHSG-TatB^EC^ (pink triangles), BØD containing pHSG-TatB^CG^ (purple asterisks), JARV15 (Δ*tatA/E*) containing pHSG575 (blue diamonds), and JARV15 containing pHSG-TatB^CG^ (red open pentagons) were analyzed for SDS sensitivity using the assay described by Ize et al. [40]. The cells were grown for 3 h in LB medium containing 100 μM IPTG in the presence of various SDS concentrations as outlined in the Material and Methods section. 100% survival is defined as the optical density of each strain after 3 h growth in LB medium without SDS. The experiments were performed in triplicate using biologically independent replicates and the respective standard deviations are indicated.

*E*. *coli tat* mutants possess pleiotropic cell envelope defects that are caused by the mislocalization of two Tat-dependent periplasmic amidases (AmiA and AmiC) that are involved in cell wall turnover [40]. Due to this fact, the *E*. *coli tat* mutants are sensitive to the presence of sodium dodecyl sulfate (SDS), unless they are complemented with a *tat* gene that allows for the formation of a functionally active Tat translocase [41]. To investigate whether the *C*. *glutamicum* TatB can indeed perform the specialized functions associated with the *E*. *coli* TatB, we tested whether the *C*. *glutamicum* TatB can complement the *E*. *coli* Δ*tatB* mutant strain BØD [17]. Following the protocol established by Ize et al. [40], the respective cells were grown in the presence of various SDS concentrations and the % survival of each strain was determined for the SDS concentrations tested (Fig 6). In contrast to the MC4100 wild-type strain (black squares), the uncomplemented Δ*tatB* mutant (green circles) is highly sensitive to SDS. As expected, SDS resistance of BØD could be restored by complementation with plasmid-encoded *E*. *coli* TatB (pink triangles). Likewise, restoration of SDS resistance was observed when the *C*. *glutamicum* TatB was expressed in BØD (purple asterisks), clearly demonstrating that the heterologous TatB can fulfill the dedicated function of the *E*. *coli* TatB protein. In contrast, expression of the *C*. *glutamicum* TatB from plasmid pHSG-TatB^CG^ in the Δ*tatA/E* mutant JARV15 [11] did not result in the formation of a functional Tat translocase, since the corresponding strain (red open pentagons) showed the same SDS sensitivity as the uncomplemented Δ*tatA/E* mutant (blue diamonds). These results strongly suggest that the *C*. *glutamicum* TatB protein in fact is a bona fide TatB protein that is functionally equivalent to the TatB proteins present in TatABC-type Tat translocases.

## Discussion

In the present study, we have reexamined the requirement of TatB for Tat-dependent protein translocation in *C*. *glutamicum*. Our results suggest that TatB very likely is strictly needed for the correct inner membrane assembly of a physiologically important Tat substrate (i.e. the Rieske iron-sulfur protein QcrA). In addition, we have found that TatB, like TatA and TatC, is essential for the secretion of a Tat-dependent heterologous model protein (PhoD^CG^-GFP) into the culture supernatant. Our findings strongly support the view that a TatABC-type translocase operates in *C*. *glutamicum* and that the individual respective translocase subunits perform the same distinct functions as their corresponding subunits in the well characterized prototype TatABC translocase of *E*. *coli*.

So far, it was unknown what causes the severe growth defect of *C*. *glutamicum* strains that lack a functional Tat translocase. Our results clearly show that the mislocalization of the carboxyl-terminal iron-sulfur cofactor-containing domain of the QcrA Rieske protein is at least one of the major reasons for the poor growth of *C*. *glutamicum tat* mutants. Such a conclusion is supported by several findings: First, the *C*. *glutamicum* Δ*qcrA* mutant and all *C*. *glutamicum* mutant strains that lack a functional Tat translocase show an almost identical growth defect under the fully aerobic conditions in the BioLector micro reactor cultivation device. Second, the replacement of the twin-arginine residues of the internal Tat motif preceding the third transmembrane segment by a twin-lysine pair completely abolished the ability of plasmid-encoded QcrA to complement a Δ*qcrA* mutant strain, being strong evidence that the translocation of the carboxyl-terminal iron-sulfur domain to the *trans*-side of the plasma membrane is a Tat-dependent step. Finally, only the growth defect of a Δ*qcrA* single mutant, but not that of a Δ*qcrA* Δ*tatB* double mutant could be complemented by plasmid-encoded QcrA, strongly supporting the view that a functional Tat system is required for correct QcrA membrane assembly. However, despite our data show that the defective main branch of the respiratory chain is a major reason for the severe growth defect of *C*. *glutamicum tat* mutants, we cannot completely exclude the possibility that the mislocalization of (an)other Tat substrate(s) also has negative impacts on growth that is overshadowed by the dominant effect caused by the mislocalization of QcrA.

In principle, the iron-sulfur cofactor-containing domain of a bacterial QcrA protein can be exported also by a natural bona fide TatAC minimal Tat translocase, as has recently been demonstrated for *B*. *subtilis* [42–43]. For *C*. *glutamicum*, our results however indicate that, under fully aerobic conditions, a complete TatABC translocase is required for QcrA assembly and, based on this, for unimpaired growth. Our suggestion that TatB very likely is required for QcrA localization is, to a great extent, based on our finding that a Δ*qcrA* Δ*tatB* double mutant cannot be complemented by plasmid-encoded QcrA. However, a hypothetical scenario that might have misled us with respect to such a conclusion cannot completely be excluded and is discussed in what follows. In such a scenario, besides QcrA one or more physiologically important Tat substrates would exist whose mislocalization has an identically severe negative impact on growth as the mislocalization of QcrA. Furthermore, such a scenario would require that both negative effects on growth, the one caused by the mislocalization of QcrA and the one caused by the mislocalization of the additional hypothetical Tat substrate(s), are not additive since, in the QcrA-complemented Δ*qcrA* Δ*tatB* double mutant, not even a partial improvement of its growth was observed in comparison to the uncomplemented strain. Our conclusion with respect to the TatB requirement for correct QcrA localization would only be incorrect if, against all expectations, QcrA would be special in that it solely relies on TatA and TatC for its membrane assembly, whereas in contrast the localization of the additional hypothetical physiologically important Tat substrate(s), and also the secretion of the heterologous TorA/PhoD^CG^-GFP model proteins, strictly requires the presence of TatB (since a *tatB* mutant shows the same severe growth defect as a *tatAC* mutant and no TorA/PhoD^CG^-GFP is secreted by the *tatB* mutant). To the best of our knowledge, such an extreme variation in the utilization of Tat components between different Tat substrates or, in other words, the co-existence and substrate-dependent differential use of a TatAC and a TatABC translocase, has never been observed in any TatABC-containing microorganism. With that said, we think that it is very likely that TatB (along with TatA and TatC) is required for proper QcrA assembly.

With respect to the effect of a *tatB* deletion on the growth of *C*. *glutamicum*, our results which show that TatB is of equal importance as TatAC for growth under aerobic conditions significantly differ from the results of Kikuchi et al. [30] which reported that deletion of *tatA* or *tatC*, but not of *tatB* resulted in a strong growth defect of the corresponding *C*. *glutamicum tat* mutant strains. The different behavior of the two Δ*tatB* mutants with respect to growth is also manifested in their different ability for the secretion of GFP model proteins. All our *tat* mutant strains tested (i.e. Δ*tatB*, Δ*tatAC*, and Δ*tatA/E*) showed a complete defect in the secretion of PhoD^CG^-GFP (Fig 3) and TorA-GFP (S1 Fig) into the culture supernatant. In contrast, a significant secretion of TorA-GFP (i.e. 9% of the wild-type strain level) into the culture supernatant of their Δ*tatB* mutant strain was observed by Kikuchi et al. [30]. Taken together, it seems that the two Δ*tatB* mutant strains possess different residual activities with respect to the Tat-dependent translocation of a native protein (i.e. QcrA) and also of heterologous model proteins (i.e. TorA-GFP/PhoD^CG^-GFP), respectively.

We can only speculate about the possible reason(s) for the observed differences. The TatA protein of *C*. *glutamicum* ATCC13869, the parent strain used in the study of Kikuchi et al. [30], is 12 amino acid residues longer than the TatA protein of *C*. *glutamicum* ATCC13032 which was used as the parent strain in our present study. The two TatA proteins show 88% identity in the overlapping regions and some of the amino acid differences can be found in the amino-terminal region [30]. Since previous results have shown that *E*. *coli* TatA can be converted by even a single amino acid alteration within the amino-terminal region into a bifunctional TatA protein that can take over the function of TatB to a significant degree [19], one possible explanation might be that the TatA proteins from the two *C*. *glutamicum* strains differ with respect to their evolutionary remnant bifunctionality. If so, then the *C*. *glutamicum* ATCC13869 Δ*tatB* mutant might possess a higher residual transport activity than *C*. *glutamicum* ATCC13032 Δ*tatB* because its TatA protein can more efficiently compensate for the absence of TatB compared to its *C*. *glutamicum* ATCC13032 counterpart. Another point that should be kept in mind is that our parent strain *C*. *glutamicum* ATCC13032 is an untreated *C*. *glutamicum* wild-type strain [44]. In contrast, the actual strain that has been used in the study of Kikuchi et al. [30] for the construction and subsequent characterization of the various *tat* deletion mutants was YDK010, an industrial *C*. *glutamicum* ATCC13869 derivative that has been extensively mutagenized by N-methyl-N-nitro-N-nitrosoguanidine (MNNG) followed by a screening for improved secretory production of proteins [45]. An alternative explanation therefore might be that in YDK010 a bifunctional TatA protein (TatA*) has been created during MNNG mutagenesis of the ATCC13869 parental strain that, due to an MNNG-induced mutation, bypasses to some degree the essential requirement of TatB for unimpaired growth under aerobic conditions. Besides this, also mutations in one of the other remaining *tat* genes in the YDK010 Δ*tatB* mutant (i.e. *tatE* or *tatC*) that cause a suppression of the growth defect of the YDK010 Δ*tatB* mutant strain can also not be completely excluded.

Finally, our finding that the *C*. *glutamicum* TatB can functionally complement an *E*. *coli* Δ*tatB*, but not an *E*. *coli* Δ*tatA/E* mutant strongly suggests that the *C*. *glutamicum* TatB is in fact a bona fide TatB protein that, together with TatA(E) and TatC performs a distinct function in a TatABC-type translocase also in its native surrounding. Together with results from other Actinobacteria containing a *tatAC* operon and an additional *tatB* gene located elsewhere on the chromosome, such as *M*. *tuberculosis* [25], *S*. *lividans* [26–27], or *S*. *coelicolor* [28–29, 41], the combined data of our present study for *C*. *glutamicum* clearly strengthen the view that a TatABC-type translocase is the physiologically relevant functional unit for Tat-dependent protein translocation in most, if not all, members of this special class of microorganisms.

## Materials and Methods

### Bacterial strains, media, and growth conditions

The bacterial strains used in this study are listed in Table 1. *E*. *coli* strains were grown at 37°C in LB (lysogeny broth) medium [46]. The complementation of *E*. *coli tat* mutants by plasmid-encoded Tat proteins was analyzed in LB medium containing 0.1%, 0.5%, 1%, 2%, or 4% (w/v) sodium dodecyl sulfate (SDS). *C*. *glutamicum* strains were grown in BHIS medium, containing 37 g/L brain heart infusion (Difco) and 91 g/L sorbitol, at 30°C. If required, isopropyl-ß-D-thiogalactopyranoside (IPTG) was added at concentrations of 10 μM or 100 μM, as indicated. Antibiotic supplements were at the following concentrations: kanamycin 25 μg/ml (*C*. *glutamicum*), chloramphenicol 25 μg/ml (*E*. *coli*) and 12.5 μg/ml (*C*. *glutamicum*), respectively.

**Table 1:** Bacterial strains and plasmids used in this study.

Strains or plasmids	Relevant properties^a^	Source or reference
***E*.*coli* strains**		
MC4100	F^-^ *araD*139 Δ(*argF*-*lac*)U169 *rpsL*150 *relA*1 *fruA*25	[55]
BØD	MC4100 Δ*tatB*	[17]
JARV15	MC4100 Δ*tatA* Δ*tatE*	[11]
***C*.*glutamicum* strains**		
ATCC13032	wild-type	[44]
ATCC13032 Δ*tatAC*	Δ*tatAC*	[35]
ATCC13032 Δ*tatB*	Δ*tatB*	This study
ATCC13032 Δ*tatE*	Δ*tatE*	This study
ATCC13032 Δ*tatA/E*	Δ*tatA/E*	This study
ATCC13032 Δ*qcrA*	Δ*qcrA*	This study
ATCC13032 Δ*qcrA* Δ*tatB*	Δ*qcrA* Δ*tatB*	This study
***E*. *coli*** plasmids		
pHSG575	pSC101 replicon, *lacZ*α+ Cm^R^	[54]
pHSG-TatB^EC^	pHSG575 containing the *E*. *coli tatB* gene	This study
pHSG-TatB^CG^	pHSG575 containing the *C*. *glutamicum tatB* gene	This study
***C*. *glutamicum*** plasmids		
pEKEx2	*C*. *glutamicum*/*E*. *coli* shuttle vector for regulated gene expression, pBL1 *oriV*_*C*.*glutamicum*_, pUC18 *oriV*_*E*.*coli*_, P_*tac*_, *lac*I^q^, Km^R^	[52]
pCGPhoD^CG^-GFP	pEKEx2 containing a gene encoding PhoD^CG^-GFP	[35]
pCGTorA-GFP	pEKEx2 containing a gene encoding TorA-GFP	[35]
pEKEx2-QcrA	pEKEx2 containing the *C*. *glutamicum qcrA* gene	This study
pEKEx2-QcrA_KK_	pEKEx2-QcrA (R159K,R160K)	This study
pEC-XC99E	pGA1 mini replicon, P*trc*, *lacI* ^q^, Cm^R^	[53]
pEC-TatB^CG^	pEC-XC99E containing the *C*. *glutamicum tatB* gene	This study
pK19mobsacB	Vector for allelic exchange in *C*. *glutamicum*, pK18 *oriV*_*E*.*coli*_, *oriT*, *mob*, *sacB*, *lacZ*α, Km^R^	[49]
pK19mobsacBΔ*qcrA*	pK19mobsacB containing the flanking regions of *C*. *glutamicum qcrA*	This study
pK19mobsacBΔ*tatE*	pK19mobsacB containing the flanking regions of *C*. *glutamicum tatE*	This study
pK19mobsacBΔ*tatA*	pK19mobsacB containing the flanking regions of *C*. *glutamicum tatA*	This study
pCRD206	pCGR2 replicon (ts), sacB, Km^R^	[51]
pCRD206Δ*tatB*	pCRD206 containing the flanking regions of *C*. *glutamicum tatB*	This study

^a^Km^R^, kanamycin resistance; Cm^R^, chloramphenicol resistance.

Growth curves of *C*. *glutamicum* strains were determined in 48-well FlowerPlates (mp2-labs, Aachen/D) using the BioLector micro reactor cultivation device (m2p-labs, Aachen/D) that allows the online monitoring of biomass formation [36]. 5 ml BHIS medium were inoculated with a fresh colony of the respective strains from a BHIS agar plate and grown overnight as the first preculture at 30°C and 170 rpm. Subsequently, 20 ml BHIS medium in a 4-chicane Erlenmeyer flask were inoculated from the first preculture to an OD_600_ of 0.5 and grown overnight at 30°C and 120 rpm. The OD_600_ of the respective second precultures was measured and equal amounts of cells from the different strains were centrifuged and subsequently resuspended in fresh BHIS medium to an OD_600_ of 0.5. 750 μl of the respective cultures were transferred into the wells of the 48-well FlowerPlates that were subsequently cultivated for 48 h in the BioLector at 30°C, 1100 rpm under constant 85% relative humidity. The production of biomass was determined as the backscattered light intensity of sent light with a wavelength of 620 nm (signal gain factor 20). Measurement intervals were set to 15 min. In each of the experiments shown in Figs 1, 2, 4, and 5, the growth curves of all strains that are shown in the respective figure were determined in biologically independent triplicates on the same FlowerPlate.

### Strain constructions

Routine methods such as PCR, DNA restriction, and ligation were performed using standard protocols [47]. Oligonucleotides used as PCR primers are listed in S1 Table. Chromosomal DNA of *C*. *glutamicum* was prepared as described previously [48]. The construction of *C*. *glutamicum* ATCC13032 Δ*tatAC* has been described previously [35]. *C*. *glutamicum* ATCC13032 Δ*tatA/E* was constructed by deleting *tatE* and *tatA* in a stepwise manner using the pK19mobsacB system which carries the counter-selectable marker *sacB* [49]. First, a deletion of *tatE* in *C*. *glutamicum* ATCC13032 wild-type was constructed by generating two DNA fragments containing approximately 480 bp of upstream and downstream sequences of the *tatE* gene using chromosomal DNA of *C*. *glutamicum* ATCC13032 as template together with primers del_tatEup-f and del_tatEup-r or primers del_tatEdn-f and del_tatEdn-r, respectively. Both PCR products were purified and used as a template in a cross-over PCR reaction using primers del_tatEup-f and del_tatEdn-r to generate a fused DNA fragment that contained the up- and downstream sequences of *tatE*, but lacks the *tatE* structural gene. The corresponding PCR fragment was digested with *Sal*I and *Bam*HI and ligated into *Sal*I/*Bam*HI-digested pK19mobsacB, yielding pK19mobsacBΔ*tatE*. For deletion of the chromosomal *tatE* structural gene, pK19mobsacBΔ*tatE* was introduced into *C*. *glutamicum* ATCC13032 by electroporation [50]. Cells that had integrated the plasmid into the chromosome via homologous recombination were selected on plates containing kanamycin. A second homologous recombination event leading to the loss of the *sacB* gene (via excision of the integrated plasmid) was positively selected on BHIS agar plates containing 10% sucrose. Colonies were analyzed for the presence or absence of the *tatE* gene by colony PCR using primers deltaE-f and deltaE-r. One of the isolates that contained the desired chromosomal deletion of the *tatE* structural gene was designated *C*. *glutamicum* ATCC13032 Δ*tatE*. Next, a deletion of *tatA* in *C*. *glutamicum* ATCC13032 Δ*tatE* was constructed by generating two DNA fragments containing approximately 520 bp upstream and downstream sequences of the *tatA* structural gene using chromosomal DNA of *C*. *glutamicum* ATCC13032 as template together with primers del_tatAup-f and del_tatAup-r or primers del_tatAdn-f and del_tatAdn-r, respectively. Both PCR products were purified and used as a template in a cross-over PCR reaction using primers del_tatAup-f and del_tatAdn-r to generate a fused DNA fragment that contained the up- and downstream sequences of *tatA*, but lacks the *tatA* structural gene. The corresponding PCR fragment was digested with *Xba*I and *Eco*RI and ligated into *Xba*I/*Eco*RI-digested pK19mobsacB, yielding pK19mobsacBΔ*tatA*. For deletion of the chromosomal *tatA* structural gene, pK19mobsacBΔ*tatA* was introduced into *C*. *glutamicum* ATCC13032 Δ*tatE* by electroporation and further processed as described above. Colonies were analyzed for the presence or absence of the *tatA* gene by colony PCR using primers CgDel-for and CgDel-rev. One of the isolates that besides the *tatE* deletion also contained the desired chromosomal deletion of the *tatA* structural gene was designated *C*. *glutamicum* ATCC13032 Δ*tatA/E*.

*C*. *glutamicum* ATCC13032 Δ*tatB* was constructed by deleting the *tatB* gene using the pCRD206-based system that contains a temperature-sensitive replicon and also the *sacB* gene as a counter-selectable marker [51]. Two DNA fragments containing approximately 480 bp upstream and downstream sequences of the *tatB* gene were generated using chromosomal DNA of *C*. *glutamicum* ATCC13032 as template together with primers del_tatBup-f and del_tatBup-r or primers del_tatBdn-f and del_tatBdn-r, respectively. Both PCR products were purified and used as a template in a cross-over PCR reaction using primers del_tatBup-f and del_tatBdn-r to generate a fused DNA fragment that contained the up- and downstream sequences of *tatB*, but lacks the *tatB* structural gene. The corresponding PCR fragment was digested with *Xba*I and *Sbf*I and ligated into *Xba*I/*Sbf*I-digested pCRD206, yielding pCRD206Δ*tatB*. For deletion of the chromosomal *tatB* structural gene, pCRD206Δ*tatB* was introduced into *C*. *glutamicum* ATCC13032 by electroporation and further processed as described by Okibe et al. [51]. Colonies were analyzed for the presence or absence of the *tatB* gene by colony PCR using primers deltaB-f and deltaB-r. One of the isolates that contained the desired chromosomal deletion of the *tatB* structural gene was designated *C*. *glutamicum* ATCC13032 Δ*tatB*. The C. glutamicum ATCC13032 Δ*qcrA* Δ*tatB* double mutant was constructed in an identical way by applying the pCRD206Δ*tatB* deletion vector to the *C*. *glutamicum* ATCC13032 Δ*qcrA* mutant strain (see below).

For the deletion of the *C*. *glutamicum qcrA* structural gene from the chromosome, the pK19mobsacB system was used. Two DNA fragment containing approximately 490 bp of upstream and downstream sequences of the *qcrA* gene were generated using chromosomal DNA of *C*. *glutamicum* ATCC13032 as template together with primers del_qcrAup-f and del_qcrAup-r or primers del_qcrAdn-f and del_qcrAdn-r, respectively. Both PCR products were purified and used as a template in a cross-over PCR reaction using primers del_qcrAup-f and del_qcrAdn-r to generate a fused DNA fragment that contained the up- and downstream sequences of *qcrA*, but lacks the *qcrA* structural gene. The corresponding PCR fragment was digested with *Xba*I and *Xma*I and ligated into *Xba*I/*Xma*I-digested pK19mobsacB, yielding pK19mobsacBΔ*qcrA*. For deletion of the chromosomal *qcrA* structural gene, pK19mobsacBΔ*qcrA* was introduced into *C*. *glutamicum* ATCC13032 by electroporation and further processed as described above, resulting in the strain *C*. *glutamicum* ATCC13032 Δ*qcrA*.

### Plasmid constructions

The plasmids used in this study are listed in Table 1. Oligonucleotides used as PCR primers are listed in S1 Table. All DNA manipulations followed standard procedures [47]. The correctness of all newly constructed plasmids was verified by DNA sequencing.

To allow plasmid-based expression of *C*. *glutamicum* QcrA in *C*. *glutamicum*, the *qcrA* gene was amplified by PCR using chromosomal DNA of *C*. *glutamicum* ATCC13032 as template and primers *Sal*I-qcrA-f and qcrA-*Kpn*I-r. The resulting PCR fragment was digested with *Sal*I and *Kpn*I and ligated into *Sal*I/*Kpn*I-digested pEKEx2 [52], resulting in pEKEx2-QcrA. From this plasmid, a variant (pEKEx2-QcrA_KK_) was constructed that encodes a QcrA_KK_ mutant protein in which the two conserved twin-arginine residues at positions 159 and 160 were replaced by a twin-lysine pair. The corresponding mutations were introduced into pEKEx2-QcrA via the Quick Change II site-directed mutagenesis kit (Agilent) using QC:QcrARR-KK-f and QC:QcrARR-KK-r as primers.

For plasmid-based expression of the *C*. *glutamicum* TatB in *C*. *glutamicum*, the *tatB* gene was amplified by PCR using chromosomal DNA of *C*. *glutamicum* ATCC13032 as template and primers *Sac*I-tatB-f and tatB-*Bam*HI-r. The resulting PCR fragment was digested with *Sac*I and *Bam*HI and ligated into *Sac*I/*Bam*HI-digested pEC-XC99E [53], resulting in pEC-TatB^CG^.

For plasmid-based expression of *E*. *coli* TatB in *E*. *coli tat* mutant strains, the *tatB* gene was amplified by PCR using *E*. *coli* MC4100 chromosomal DNA as template and primers *Eco*RI-tatBEc-f and EctatB-*Sal*I-r. The resulting PCR fragment was digested with *Eco*RI and *Sal*I and ligated into the *Eco*RI/*Sal*I-digested low copy number vector pHSG575 [54], resulting in plasmid pHSG-TatB^EC^.

For plasmid-based expression of *C*. *glutamicum* TatB in *E*. *coli tat* mutant strains, the *tatB* gene was amplified by PCR using *C*. *glutamicum* ATCC13032 chromosomal DNA as template and primers *Eco*RI-tatB-f and tatB-*Sal*I-r. The resulting PCR fragment was digested with *Eco*RI and *Sal*I and ligated into *Eco*RI/*Sal*I-digested pHSG575, resulting in plasmid pHSG-TatB^CG^.

### Miscellaneous procedures

For the analysis of PhoD^CG^-GFP secretion in *C*. *glutamicum* wild-type and *tat* mutant strains, the respective *C*. *glutamicum* cells containing plasmid pCGPhoD^CG^-GFP were grown in BHIS medium in 4-chicane Erlenmeyer flasks at 30°C and 120 rpm to an OD_600_ of 1.0 and gene expression was induced for 6 h by adding 100 μM IPTG. Subsequently, cellular and supernatant fractions were prepared as described previously [35]. The distribution of GFP-derived polypeptides in the corresponding fractions was analyzed by sodium dodecyl sulfate-polyacrylamide gel electrophoresis (SDS-PAGE) and Western blotting using GFP-specific antibodies. Western blotting was performed using the ECL Western blotting detection kit (GE Healthcare) according to the manufacturer’s instructions. The chemiluminescent protein bands were recorded using the Fuji LAS-3000 Mini CCD camera and image analyzing system using the software AIDA 4.15 (Raytest).

*E*. *coli tat* mutants are sensitive to the presence of SDS unless they are complemented by functional homologous or heterologous *tat* genes [41]. The complementation of *E*. *coli tat* mutants by plasmid-encoded TatB proteins from *E*. *coli* or *C*. *glutamicum* was analyzed by the assay described by Ize et al. [40]. Overnight cultures of the strains were diluted to an OD_600_ of 0.05 with LB medium containing either 0.1%, 0.5%, 1%, 2% or 4% SDS (w/v) and grown aerobically for 180 min at 37°C and 250 rpm. Subsequently, the OD_600_ of each culture was measured. 100% survival is defined as the OD_600_ of each strain after 180 min growth in LB medium without SDS.

## Supporting Information

S1 Fig Analysis of TorA-GFP secretion in *C*. *glutamicum* wild-type and *tat* mutant strains. Cultures of *C*. *glutamicum* strains expressing the Tat-dependent TorA-GFP model protein [35] were fractionated into cells (C) and supernatant (S). Samples of the fractions corresponding to an equal number of cells (i.e. an OD_600_ of 1.0) were subjected to SDS-PAGE and immunoblotting using GFP-specific antibodies. The following strains were analyzed: *C*. *glutamicum* wild-type (wt) containing the empty vector pEKEx2 as negative control (lanes 1 and 2), *C*. *glutamicum* wild-type (wt) containing plasmid pCGTorA-GFP (lanes 3 and 4) and the pCGTorA-GFP-containing *C*. *glutamicum* mutant strains Δ*tatAC* (lanes 5 and 6), Δ*tatB* (lanes 7 and 8), and Δ*tatA/E* (lanes 9 and 10). p: TorA-GFP precursor; asterisk: cytosolic degradation product; m: mature-sized GFP protein.
(TIF)

S2 Fig Uncropped image of Western blot shown in Fig 3. (TIF)

S3 Fig Uncropped image of Western blot shown in S1 Fig
(TIF)

S1 Table Primers used in this study. (DOCX)

## References

[1] Desvaux M, Parham NJ, Scott-Tucker A, Henderson IR. The general secretory pathway: a general misnomer? Trends Microbiol. 2004;12: 306–309. 15223057 10.1016/j.tim.2004.05.002

[2] Denks K, Vogt A, Sacchelaru I, Petriman NA, Kudva R, Koch HG. The Sec translocon mediated protein transport in prokaryotes and eukaryotes. Mol Membr Biol. 2014;31: 58–84. 10.3109/09687688.2014.907455 24762201

[3] Palmer T, Berks BC. The twin-arginine translocation (Tat) protein export pathway. Nat Rev Microbiol. 2012;10: 483–496. 10.1038/nrmicro2814 22683878

[4] Berks BC. A common export pathway for proteins binding complex redox cofactors? Mol Microbiol. 1996;22: 393–404. 8939424 10.1046/j.1365-2958.1996.00114.x

[5] Stanley NR, Palmer T, Berks BC. The twin arginine consensus motif of Tat signal peptides is involved in Sec-independent protein targeting in *Escherichia coli* . J Biol Chem. 2000;275: 11591–11596. 10766774 10.1074/jbc.275.16.11591

[6] Mendel S, McCarthy A, Barnett JP, Eijlander RT, Nenninger A, Kuipers OP, et al The *Escherichia coli* TatABC system and a *Bacillus subtilis* TatAC-type system recognise three distinct targeting determinants in twin-arginine signal peptides. J Mol Biol. 2008;375: 661–672. 18036542 10.1016/j.jmb.2007.09.087

[7] Lausberg F, Fleckenstein S, Kreutzenbeck P, Fröbel J, Rose P, Müller M, et al Genetic evidence for a tight cooperation of TatB and TatC during productive recognition of twin-arginine (Tat) signal peptides in *Escherichia coli* . PLoS ONE. 2012;7(6): e39867 10.1371/journal.pone.0039867 22761916 PMC3383694

[8] Sutcliffe IC. A phylum level perspective on bacterial cell envelope architecture. Trends Microbiol. 2010;18: 464–470. 10.1016/j.tim.2010.06.005 20637628

[9] Desvaux M, Hebraud M, Talon R, Henderson IR. Secretion and subcellular localizations of bacterial proteins: a semantic awareness issue. Trends Microbiol. 2009;17: 139–145. 10.1016/j.tim.2009.01.004 19299134

[10] Chagnot C, Zorgani MA, Astruc T, Desvaux M. Proteinaceous determinants of surface colonization in bacteria: bacterial adhesion and biofilm formation from a protein secretion perspective. Front Microbiol. 2013;4: 303 10.3389/fmicb.2013.00303 24133488 PMC3796261

[11] Sargent F, Bogsch EG, Stanley NR, Wexler M, Robinson C, Berks BC, et al Overlapping functions of components of a bacterial Sec-independent protein export pathway. EMBO J. 1998;17: 3640–3650. 9649434 10.1093/emboj/17.13.3640 PMC1170700

[12] Jack RL, Sargent F, Berks BC, Sawers G, Palmer T. Constitutive expression of *Escherichia coli tat* genes indicates an important role for the twin-arginine translocase during aerobic and anaerobic growth. J Bacteriol. 2001;183: 1801–1804. 11160116 10.1128/JB.183.5.1801-1804.2001 PMC95070

[13] Alami M, Lüke I, Deitermann S, Eisner G, Koch HG, Brunner J, et al Differential interaction between a twin-arginine signal peptide and its translocase in *Escherichia coli* . Mol Cell. 2003;12: 937–946. 14580344 10.1016/s1097-2765(03)00398-8

[14] Mori H, Cline K. A twin arginine signal peptide and the pH gradient trigger reversible assembly of the thylakoid ΔpH/Tat translocase. J Cell Biol. 2002;157: 205–210. 11956224 10.1083/jcb.200202048 PMC2199252

[15] Gohlke U, Pullan L, McDevitt CA, Porcelli I, de Leeuw E, Palmer T, et al The TatA component of the twin-arginine protein transport system forms channel complexes of variable diameter. Proc Natl Acad Sci USA. 2005;102: 10482–10486. 16027357 10.1073/pnas.0503558102 PMC1180781

[16] Brüser T, Sanders C. An alternative model of the twin arginine translocation system. Microbiol Res. 2003;158: 7–17. 12608575 10.1078/0944-5013-00176

[17] Sargent F, Stanley NR, Berks BC, Palmer T. Sec-independent protein translocation in *Escherichia coli*. A distinct and pivotal role for the TatB protein. J Biol Chem. 1999;274: 36073–36082. 10593889 10.1074/jbc.274.51.36073

[18] Jongbloed JDH, Grieger U, Antelmann H, Hecker M, Nijland R, Bron S, et al Two minimal Tat translocases in *Bacillus* . Mol Microbiol. 2004;54: 1319–1325. 15554971 10.1111/j.1365-2958.2004.04341.x

[19] Blaudeck N, Kreutzenbeck P, Müller M, Sprenger GA, Freudl R. Isolation and characterization of bifunctional *Escherichia coli* TatA mutant proteins that allow efficient Tat-dependent protein translocation in the absence of TatB. J Biol Chem. 2005;280: 3426–3432. 15557327 10.1074/jbc.M411210200

[20] Jongbloed JDH, van der Ploeg R, van Dijl JM. Bifunctional TatA subunits in minimal Tat protein translocases. Trends Microbiol. 2006;14: 2–4. 16303306 10.1016/j.tim.2005.11.001

[21] Barnett JP, Eijlander RT, Kuipers OP, Robinson C. A minimal Tat system from a Gram-positive organism. A bifunctional TatA subunit participates in discrete TatAC and TatA complexes. J Biol Chem. 2008;283: 2534–2542. 18029357 10.1074/jbc.M708134200

[22] Goosens VJ, Monteferrante CG, van Dijl JM. The Tat system of Gram-positive bacteria. Biochim Biophys Acta. 2014;1843: 1698–1706. 10.1016/j.bbamcr.2013.10.008 24140208

[23] Scherr N, Nguyen L. *Mycobacterium* versus *Streptomyces*—we are different, we are the same. Curr Opin Microbiol. 2009;12: 699–707. 10.1016/j.mib.2009.10.003 19880345

[24] Simone D, Bay DC, Leach T, Turner RJ. Diversity and evolution of bacterial twin arginine translocase protein, TatC, reveals a protein secretion system that is evolving to fit its environmental niche. PLoS ONE. 2013;8(11): e78742 10.1371/journal.pone.0078742 24236045 PMC3827258

[25] Saint-Joanis B, Demangel C, Jackson M, Brodin P, Marsollier L, Boshoff H, et al Inactivation of Rv2525c, a substrate of the twin arginine translocation (Tat) system of *Mycobacterium tuberculosis*, increases β-lactam susceptibility and virulence. J Bacteriol. 2006;188: 6669–6679. 16952959 10.1128/JB.00631-06 PMC1595485

[26] Schaerlaekens K, Schierova M, Lammertyn E, Geukens N, Anné J, van Mellaert L. Twin-arginine translocation pathway in *Streptomyces lividans* . J Bacteriol. 2001;183: 6727–6732. 11698358 10.1128/JB.183.23.6727-6732.2001 PMC95510

[27] De Keersmaeker S, Van Mellaert L, Lammertyn E, Vrancken K, Anne J, Geukens N. Functional analysis of TatA and TatB in *Streptomyces lividans* . Biochem Biophys Res Commun. 2005;335: 973–982. 16111662 10.1016/j.bbrc.2005.07.165

[28] Widdick DA, Dilks K, Chandra G, Bottrill A, Naldrett M, Pohlschröder M, et al The twin-arginine translocation pathway is a major route of protein export in *Streptomyces coelicolor* . Proc Natl Acad Sci USA. 2006;103: 17927–17932. 17093047 10.1073/pnas.0607025103 PMC1693849

[29] Willemse J, Ruban-Osmialowska B, Widdick D, Celler K, Hutchings MI, van Wezel GP, et al Dynamic localization of Tat protein transport machinery components in *Streptomyces coelicolor* . J Bacteriol. 2012;194: 6272–6281. 10.1128/JB.01425-12 23002216 PMC3486365

[30] Kikuchi Y., Date M, Itaya H, Matsui K, Wu LF. Functional analysis of the twin-arginine translocation pathway in *Corynebacterium glutamicum* ATCC 13869. Appl Environ Microbiol. 2006;72: 7183–7192. 16997984 10.1128/AEM.01528-06 PMC1636197

[31] Bott M, Niebisch A. The respiratory chain of *Corynebacterium glutamicum* . J Biotechnol. 2003;104: 129–153. 12948635 10.1016/s0168-1656(03)00144-5

[32] Keller R, de Keyzer J, Driessen AJM, Palmer T. Co-operation between different targeting pathways during integration of a membrane protein. J Cell Biol. 2012;199: 303–315. 10.1083/jcb.201204149 23045547 PMC3471235

[33] Hopkins A, Buchanan G, Palmer T. Role of the twin arginine protein transport pathway in the assembly of the *Streptomyces coelicolor* cytochrome *bc* _*1*_ complex. J Bacteriol. 2014;196: 50–59. 10.1128/JB.00776-13 24142258 PMC3911139

[34] Koch-Koerfges A, Pfelzer N, Platzen L, Oldiges M, Bott M. Conversion of *Corynebacterium glutamicum* from an aerobic respiring to an aerobic fermenting bacterium by inactivation of the respiratory chain. Biochim Biophys Acta. 2013;1827: 699–708. 10.1016/j.bbabio.2013.02.004 23416842

[35] Meissner D, Vollstedt A, van Dijl JM, Freudl R. Comparative analysis of twin-arginine (Tat)-dependent protein secretion of a heterologous model protein (GFP) in three different Gram-positive bacteria. Appl Microbiol Biotechnol. 2007;76: 633–642. 17453196 10.1007/s00253-007-0934-8

[36] Samorski M, Müller-Newen G, Büchs J. Quasi-continuous combined scattered light and fluorescence measurements: a novel measurement technique for shaken microtiter plates. Biotechnol Bioeng. 2005;92: 61–68. 15988771 10.1002/bit.20573

[37] Niebisch A, Bott M. Molecular analysis of the cytochrome *bc* _*1*_ *-aa* _*3*_ branch of the *Corynebacterium glutamicum* respiratory chain containing an unusual diheme cytochrome c_1_ . Arch Microbiol. 2001;175: 282–294. 11382224 10.1007/s002030100262

[38] Cristobal S, de Gier JW, Nielsen H, von Heijne G. Competition between Sec- and TAT-dependent protein translocation in *Escherichia coli* . EMBO J. 1999;18: 2982–2990. 10357811 10.1093/emboj/18.11.2982 PMC1171380

[39] Berks BC, Palmer T, Sargent F. The Tat protein translocation pathway and its role in microbial physiology. Adv Microb Physiol. 2003;47: 187–254. 14560665 10.1016/s0065-2911(03)47004-5

[40] Ize B, Stanley NR, Buchanan G, Palmer T. Role of the *Escherichia coli* Tat pathway in outer membrane integrity. Mol Microbiol. 2003;48: 1183–1193. 12787348 10.1046/j.1365-2958.2003.03504.x

[41] Hicks MG, Guymer D, Buchanan G, Widdick DA, Caldelari I, Berks BC, et al Formation of functional Tat translocases from heterologous components. BMC Microbiol. 2006;6: 64 16854235 10.1186/1471-2180-6-64 PMC1550398

[42] Goosens VJ, Otto A, Glasner C, Monteferrante CC, van der Ploeg R, Hecker M, et al Novel twin-arginine translocation pathway-dependent phenotypes of *Bacillus subtilis* unveiled by quantitative proteomics. J Proteome Res. 2013;12: 796–807. 10.1021/pr300866f 23256564

[43] Goosens VJ, Monteferrante CG, van Dijl JM. Co-factor insertion and disulfide bond requirements for twin-arginine translocase-dependent export of the *Bacillus subtilis* Rieske protein QcrA. J Biol Chem. 2014;289: 13124–13131. 10.1074/jbc.M113.529677 24652282 PMC4036324

[44] Kinoshita S, Udaka S, Shimono M. Studies on amino acid fermentation. Part I. Production of L-glutamic acid by various microorganisms. J Gen Appl Microbiol. 1957;3: 193–205. 15965888

[45] Kikuchi Y, Date M, Umezawa Y, Yokoyama K, Heima H, Matsui H. Method for the secretion and production protein. International Patent Cooperation Treaty Patent. 2002;WO2002/081694.

[46] Bertani G. Studies on lysogenesis. I. The mode of phage liberation by lysogenic *Escherichia coli* . J Bacteriol. 1951;62: 293–300. 14888646 10.1128/jb.62.3.293-300.1951 PMC386127

[47] Sambrook J, MacCallum P, Russell D. Molecular Cloning A Laboratory Manual. 3rd ed Cold Spring Harbor NY: Cold Spring Harbor Laboratory Press; 2001.

[48] Eikmanns BJ, Thumschmitz N, Eggeling L, Lüdtke K. U.; Sahm H. Nucleotide sequence, expression and transcriptional analysis of the *Corynebacterium glutamicum gltA* gene encoding citrate synthase. Microbiology (UK). 1994;140: 1817–1828. 10.1099/13500872-140-8-1817 7522844

[49] Schäfer A, Tauch A, Jäger W, Kalinowski J, Thierbach G, Pühler A. Small mobilizable multi-purpose cloning vectors derived from the *Escherichia coli* plasmids pK18 and pK19: selection of defined deletions in the chromosome of *Corynebacterium glutamicum* . Gene. 1994;145: 69–73. 8045426 10.1016/0378-1119(94)90324-7

[50] van der Rest ME, Lange C, Molenaar D. A heat shock following electroporation induces highly efficient transformation of *Corynebacterium glutamicum* with xenogeneic plasmid DNA. Appl Microbiol Biotechnol. 1999;52: 541–545. 10570802 10.1007/s002530051557

[51] Okibe N, Suzuki N, Inui M, Yukawa H. Efficient markerless gene replacement in *Corynebacterium glutamicum* using a new temperature-sensitive plasmid. J Microbiol Methods. 2011;85: 155–163. 10.1016/j.mimet.2011.02.012 21362445

[52] Eikmanns BJ, Kleinertz E, Liebl W, Sahm H. A family of *Corynebacterium glutamicum* / *Escherichia coli* shuttle vectors for cloning, controlled gene expression, and promoter probing. Gene. 1991;102: 93–98. 1864513 10.1016/0378-1119(91)90545-m

[53] Kirchner O, Tauch A. Tools for genetic engineering in the amino acid-producing bacterium *Corynebacterium glutamicum* . J Biotechnol. 2003;104: 287–299. 12948646 10.1016/s0168-1656(03)00148-2

[54] Takeshita S, Sato M, Toba M, Masahashi W, Hashimoto-Gotoh T. High-copy-number and low-copy-number vectors for *lacZα*-complementation and chloramphenicol- or kanamycin-resistance selection. Gene. 1987;61: 63–74. 3327753 10.1016/0378-1119(87)90365-9

[55] Casadaban MJ. Transposition and fusion of the *lac* genes to selected promoters in *Escherichia coli* using bacteriophage lambda and Mu. J Mol Biol. 1976;104: 541–555. 781293 10.1016/0022-2836(76)90119-4

