# Supplementary figures and images for: A TatABC-Type Tat Translocase Is Required for Unimpaired Aerobic Growth of Corynebacterium glutamicum ATCC13032
Source: PLoS One. 2015 Apr 2;10(4):e0123413. doi: 10.1371/journal.pone.0123413 (PMC4383559; doi:10.1371/journal.pone.0123413)

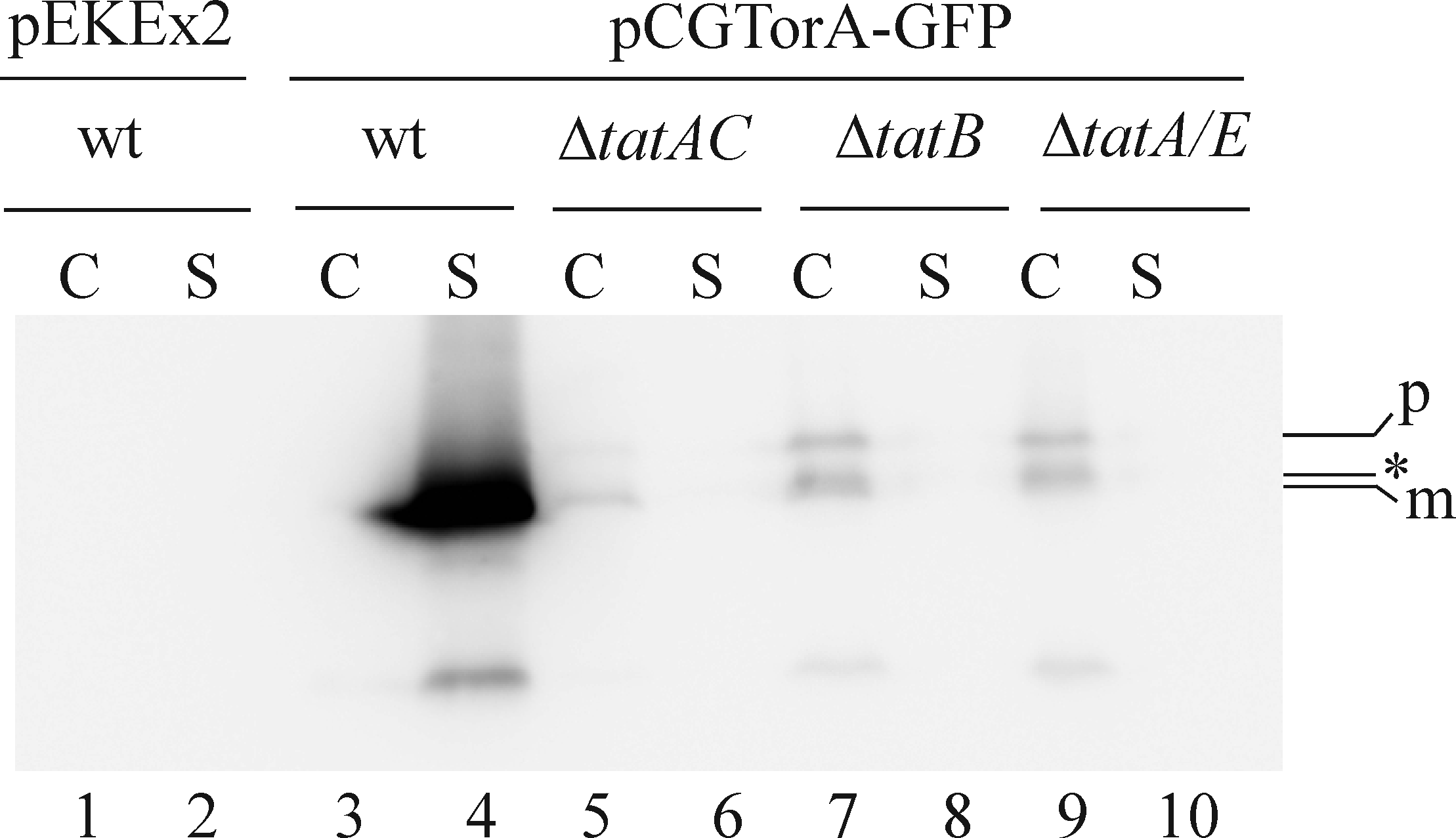

Supplement: S1 Fig — Cultures of C. glutamicum strains expressing the Tat-dependent TorA-GFP model protein [35] were fractionated into cells (C) and supernatant (S). Samples of the fractions corresponding to an equal number of cells (i.e. an OD600 of 1.0) were subjected to SDS-PAGE and immunoblotting using GFP-specific antibodies. The following strains were analyzed: C. glutamicum wild-type (wt) containing the empty vector pEKEx2 as negative control (lanes 1 and 2), C. glutamicum wild-type (wt) containing plasmid pCGTorA-GFP (lanes 3 and 4) and the pCGTorA-GFP-containing C. glutamicum mutant strains ΔtatAC (lanes 5 and 6), ΔtatB (lanes 7 and 8), and ΔtatA/E (lanes 9 and 10). p: TorA-GFP precursor; asterisk: cytosolic degradation product; m: mature-sized GFP protein. (TIF) [file pone.0123413.s001.tif]

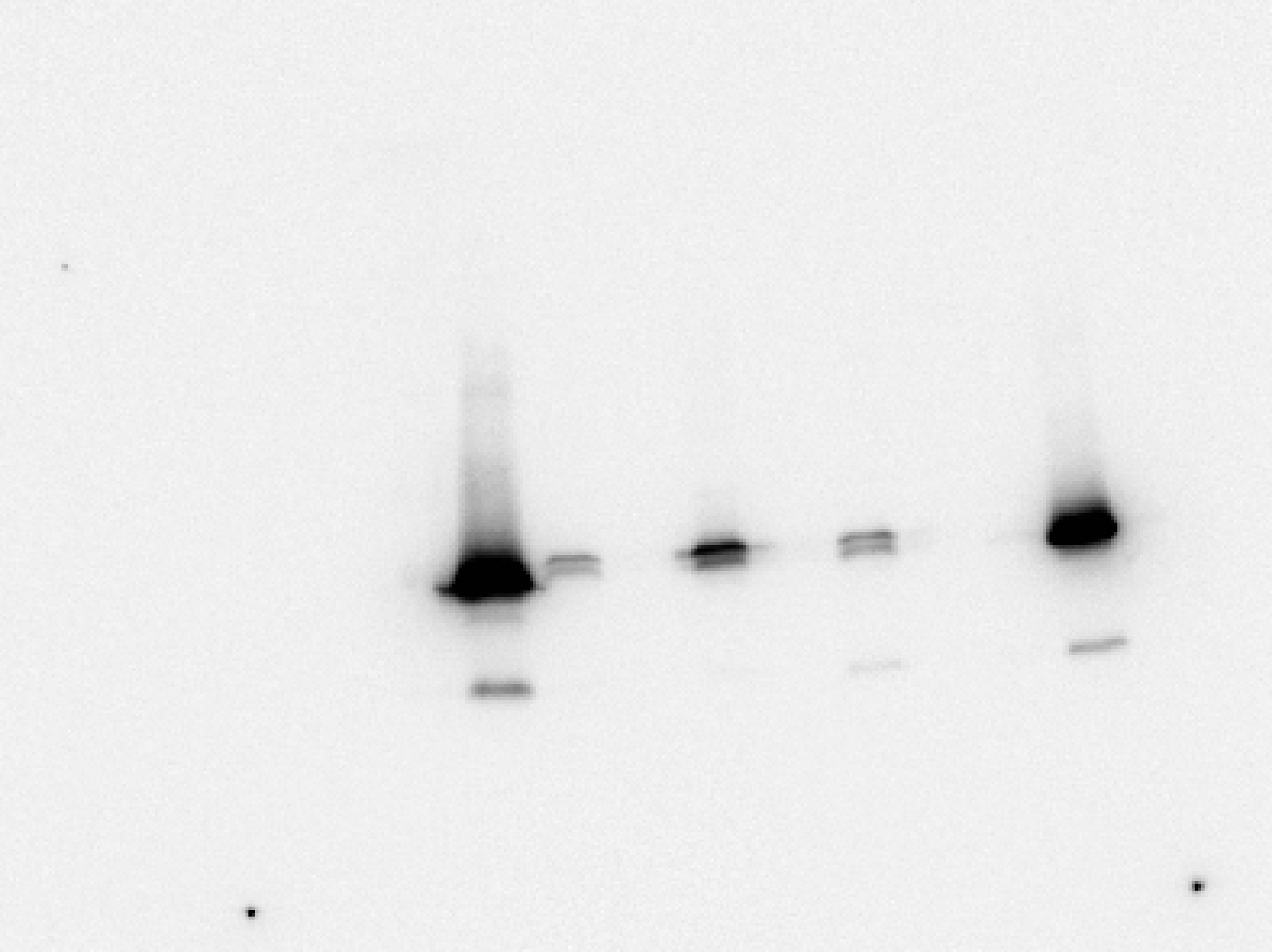

Supplement: S2 Fig — (TIF) [file pone.0123413.s002.tif]

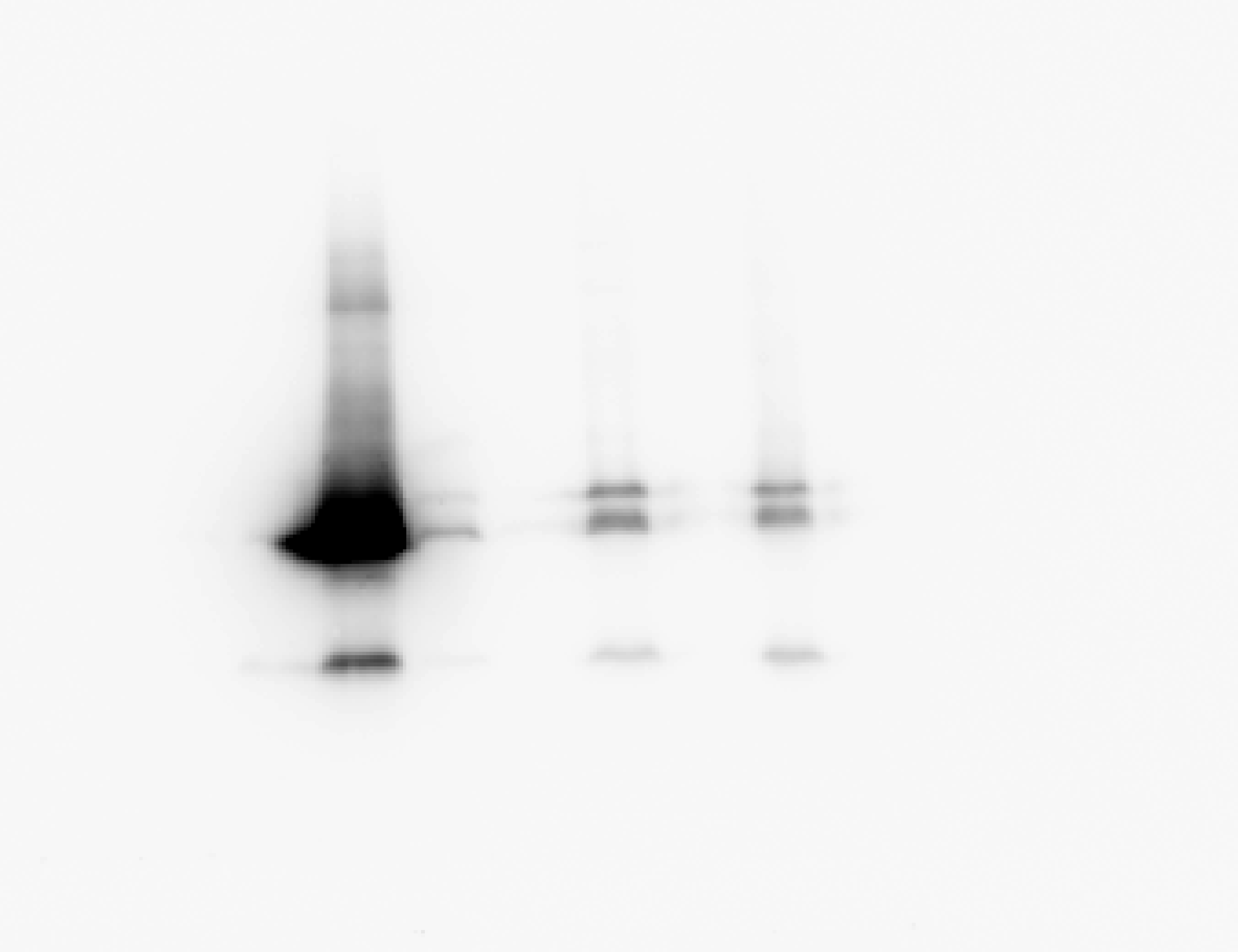

Supplement: S3 Fig — (TIF) [file pone.0123413.s003.tif]
