# Supplementary material for: A TatABC-Type Tat Translocase Is Required for Unimpaired Aerobic Growth of Corynebacterium glutamicum ATCC13032
Source: PLoS One. 2015 Apr 2;10(4):e0123413. doi: 10.1371/journal.pone.0123413 (PMC4383559; doi:10.1371/journal.pone.0123413)
Supplement: S1 Table — (DOCX) [file pone.0123413.s004.docx]

**S1 Table.** **Primers used in this study^a^**

| Primer | Sequence (5’→3’) |
| --- | --- |
| del_tatBup-f | GAGTCTAGAGGGATGTTTTGGATGTTCGAACAAAAAC |
| del_tatBup-r | CCATCCACTAAACTTAAACAACCCACGCTAGAAAACATCAGACC |
| del_tatBdn-f | TGTTTAAGTTTAGTGGATGGGTACTCAGGTGGCGTCTCTTGG |
| del_tatBdn-r | GTGCCTGCAGGCCAATTGATCCCGAATGCTGAG |
| del_tatEup-f | GAGGTCGACGTTGTTAAACATGTCCTCGGCG |
| del_tatEup-r | CCCATCCACTAAACTTAAACAAATATTTCTGCATTCCACCCTAG |
| del_tatEdn-f | TGTTTAAGTTTAGTGGATGGGCAGATCGAAAGCTCCGATCAG |
| del_tatEdn-r | GTGGGATCCGAACACTACCCCAATGGCACTGATC |
| del_tatAup-f | GCGCTCTAGAGACAGCAAAGCAGTGCACCC |
| del_tatAup-r | GACAATGATTCCAATTTCCC |
| del_tatAdn-f | GGGAAATTGGAATCATTGTCCGTCAGAACTACGAGGATCC |
| del_tatAdn-r | GCGCGAATTCGAACCATACCCACCAACGCCG |
| del_qcrAup-f | AATTCTAGAGCTAATGGCGGTGGCCCAGGACTCG |
| del_qcrAup-r | CCCCGTAACTAAACTTAAACATGTGTACTGTTTGTCGTTGTTGTTACTCAT |
| del_qcrAdn-f | TGTTTAAGTTTAGTTACGGGGGGCCCTGCATTCTGGGAGCGTAAGTCATGA |
| del_qcrAdn-r | AATCCCGGGAGTAACCCATGAAGCCTTCAGCCATACC |
| *Sal*I-qcrA-f | GAGAGTCGACGAAGGAGATATACATATGAGCAATGAGGATCTTGC |
| qcrA-*Kpn*I-r | GAGAGGTACCTCATGACTTACGCTCCCAGAATG |
| QC:QcrARR-KK-f | CTCTTGGCAGACCTCTACTCTTGGTAAGAAGAAGCTGATCATGGGACTTGCAGGTG |
| QC:QcrARR-KK-r | GAGAACCGTCTGGAGATGAGAACCATTCTTCTTCGACTAGTACCCTGAACGTCCAC |
| *Eco*RI_tatB-f | GCGCGAATTCAAGGAGATATAGATATGTTTTCTAGCGTGGGTTG |
| tatB_*Sal*I-r | GCGCGTCGACCTAAATAATATCGGTCCAAGAG |
| *Eco*RI_tatBEc-f | GCGCGAATTCAAGGAGATATAGATGTGTTTGATATCGGTTTTAGCG |
| EctatB_*Sal*I-r | GCGCGTCGACTTACGGTTTATCACTCGACGAAG |
| *Sac*I-tatB-f | GCGCGAGCTCAAGGAGATATAGATATGTTTTCTAGCGTGGGTTG |
| tatB-*Bam*HI-r | GCGCGGATCCCTAAATAATATCGGTCCAAGAG |
| deltaE-f | CAATGGCGTGACTCTTGGTG |
| deltaE-r | CGCGCTGTTCTTCTTCCTGC |
| deltaB-f | GATCGTGCAGGATCTAAAGC |
| deltaB-r | TCCAGCAATTCCTCACTGAC |
| CgDel-for | AGGCTTGATCGCATCCGAAG |
| CgDel-rev | CAGGCTCAAACAGTGGCATC |

^a^Restriction sites are underlined
